# Design, rationale, and baseline characteristics of the dapagliflozin in haemodialysis (DAPA-HD) trial

**DOI:** 10.1093/eschf/xvag104

**Published:** 2026-04-09

**Authors:** Thomas A Zelniker, Christopher Paschen, Christopher Mann, Manfred Eigner, Hildegard Hafner-Gießauf, Josef Kletzmayr, Matthias Lorenz, Bernhard Ludvik, Thomas Stulnig, Ingmar Waller, Johannes Werzowa, Sebastian Hödlmoser, Christian Hengstenberg, Rainer Oberbauer, Manfred Hecking

**Affiliations:** Division of Cardiology, Department of Medicine II, Medical University of Vienna, Waehringer Guertel 18-20, Vienna 1090, Austria; Division of Nephrology and Dialysis, Department of Medicine III, Medical University of Vienna, Waehringer Guertel 18-20, Vienna 1090, Austria; Division of Cardiology, Department of Medicine II, Medical University of Vienna, Waehringer Guertel 18-20, Vienna 1090, Austria; Favoriten Hospital (Klinik Favoriten, 1st Medical Department), Kundratsgasse 3, Wien 1100, Austria; Dialyseinstitut Gießauf GmbH, Elisabethstraße 54, Graz 8010, Austria; Donaustadt Hospital (Klinik Donaustadt, 3rd Medical Department), Langobardenstraße 122, Vienna 1220, Austria; Wiener Dialysezentrum, Kapellenweg 37, Vienna 1220, Austria; 1st Medical Department and Karl Landsteiner Institute for Obesity and Metabolism, Landstrasse Clinic, Juchgasse 25, Vienna 1030, Austria; Department of Medicine III and Karl Landsteiner Institute for Metabolic Diseases and Nephrology, Clinic Hietzing, Wolkersbergenstrasse 1, Vienna 1130, Austria; Dialyseinstitut Dr. Waller, Feldgasse 28–30, Feldbach 8330, Austria; Hanusch Hospital (Hanusch-Krankenhaus, 1st Medical Department), Heinrich-Collin-Straße 30, Vienna 1140, Austria; Division of Nephrology and Dialysis, Department of Medicine III, Medical University of Vienna, Waehringer Guertel 18-20, Vienna 1090, Austria; Division of Cardiology, Department of Medicine II, Medical University of Vienna, Waehringer Guertel 18-20, Vienna 1090, Austria; Division of Nephrology and Dialysis, Department of Medicine III, Medical University of Vienna, Waehringer Guertel 18-20, Vienna 1090, Austria; Division of Nephrology and Dialysis, Department of Medicine III, Medical University of Vienna, Waehringer Guertel 18-20, Vienna 1090, Austria

**Keywords:** Randomized controlled trial, Dapagliflozin, SGLT2i, Left ventricular hypertrophy, Dialysis, Kidney failure

## Abstract

**Introduction:**

Sodium-glucose co-transporter 2 inhibitors (SGLT2i) reduce cardiovascular events across a wide range of kidney function, including advanced stages of chronic kidney disease, but evidence for their efficacy in patients with kidney failure on haemodialysis is lacking. The underlying mechanisms remain incompletely understood, and whether cardiovascular benefits depend on residual kidney function is unknown.

**Study design:**

The Dapagliflozin in Hemodialysis (DAPA-HD) trial (NCT05179668) is an academic, multicentre, randomized, double-blind, placebo-controlled trial designed to assess the cardiovascular effects of dapagliflozin in 220 patients with kidney failure receiving haemodialysis. Stratified block randomization was based on patient-reported residual urine volume. The primary endpoint is the change in left ventricular mass indexed to body surface area using echocardiography after 6 months of treatment. A prespecified subgroup analysis will compare treatment effects between patients with residual urine output >200 ml/24 h versus ≤200 ml/24 h. Secondary endpoints include additional echocardiographic assessments, changes in biomarker concentrations, quality of life, and clinical events.

**Discussion:**

The DAPA-HD trial is the first trial specifically evaluating the cardiovascular and haemodynamic effects of dapagliflozin in patients with kidney failure receiving maintenance haemodialysis with and without residual urine outputs.

## Background

Cardiovascular disease is the leading cause of death in patients with kidney failure receiving haemodialysis.^1–3^ These patients represent one of the highest risk groups for cardiovascular morbidity and mortality, particularly due to left ventricular hypertrophy, heart failure, arrhythmias, and sudden cardiac death.^2,4,5^ Effective cardiovascular-kidney risk management in chronic kidney disease (CKD) is complicated by altered pharmacokinetics, disease-specific complications (including electrolyte disturbances, volume overload, and uraemia), and limited evidence from CKD populations.

Sodium-glucose co-transporter 2 inhibitors (SGLT2i) reduce cardiovascular events across a wide range of kidney functions, including patients with an estimated glomerular filtration rate (eGFR) as low as 20 ml/min/1.73 m^2^.^6,7^ However, patients with kidney failure requiring dialysis were systematically excluded from landmark SGLT2i trials. Prior studies have demonstrated reverse cardiac remodelling with SGLT2i in populations with type 2 diabetes, left ventricular hypertrophy, or heart failure, but whether these effects persist in patients with kidney failure requiring dialysis is unclear.^8–11^ Residual kidney function among patients receiving maintenance haemodialysis varies substantially and may influence the potential cardiovascular effects of SGLT2i. While formal quantification of residual renal clearance is complex and not routinely feasible at scale, residual urine output provides a pragmatic, patient-identifiable marker that reflects ongoing kidney activity and is widely used in clinical practice.^12^

**Figure 1:**
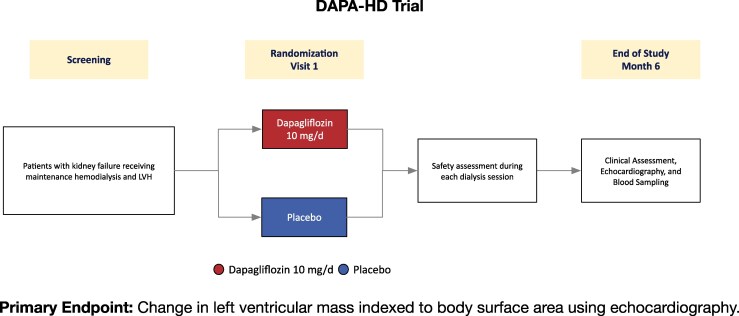
Trial design of the dapagliflozin in haemodialysis (DAPA-HD) trial. The DAPA-HD trial is an academic, investigator-initiated, multicentre, randomized, double-blind, placebo-controlled trial evaluating the effect of dapagliflozin (10 mg daily) versus placebo on the left ventricular mass indexed to body surface area using echocardiography over 6 months in patients with kidney failure receiving maintenance haemodialysis and left ventricular hypertrophy (LVH)

The Dapagliflozin in Hemodialysis (DAPA-HD) trial was designed to evaluate the cardiac effects of dapagliflozin in haemodialysis patients with prespecified stratification according to residual urine output as a pragmatic surrogate of residual kidney function. The trial tests the hypothesis that dapagliflozin can reduce left ventricular mass in this patient population and provides an opportunity to systematically assess the tolerability and safety of dapagliflozin in haemodialysis patients across the spectrum of residual kidney function (*Figure 1*).

## Methods

### Study design and population

The DAPA-HD trial (NCT05179668) is an academic, investigator-initiated, multicentre, randomized, double-blind, placebo-controlled phase 2 trial designed to evaluate the effects of the SGLT2i dapagliflozin on cardiac structure and function in patients with kidney failure receiving maintenance haemodialysis (*Figure 1*).

Patients were eligible if they were aged ≥18 years, received maintenance haemodialysis three times weekly for ≥3 months and ≤5 years, had a body mass index <45 kg/m^2^, a stable dry weight (±5 kg during the preceding 3 months), and an interventricular septal thickness >11 mm (*Table 1*). Key exclusion criteria were type 1 diabetes mellitus, a history of diabetic ketoacidosis, scheduled living-donor kidney transplantation, severe valvular heart disease, an acute coronary syndrome within the previous 30 days, known hypersensitivity or intolerance to SGLT2i, or SGLT2i therapy within the previous 6 months.

**Table 1:** Inclusion and exclusion criteria

Inclusion criteria: Age ≥18 years Maintenance haemodialysis 3×/week for ≥ 3 months and ≤5 years BMI <45 kg/m^2^ and stable weight (±5 kg [‘dry weight’]) over the preceding 3 months Interventricular septum width >11 mm **Exclusion criteria:** Treatment with SGLT2i within the last 6 months Hypersensitivity or intolerance to SGLT2 inhibitors History of Type 1 diabetes mellitus History of diabetic ketoacidosis Scheduled kidney transplant from a living donor Acute coronary syndrome during the last 30 days Severe valvular heart disease Women of childbearing potential who are unwilling or unable to use an acceptable method to avoid pregnancy for the entire study (oestrogen and/or progesterone treatment). Pregnancy Breastfeeding Substance abuse Life expectancy < 1 year Other significant disease or pathology that might predispose the patient to an unacceptable risk or interferes with the study in the opinion of the investigator.

### Treatment protocol and follow-up procedures

Eligible patients were randomized 1:1 to receive either dapagliflozin 10 mg once daily or a matching placebo for 6 months, using a stratified block randomization scheme based on residual urine volume (>200 ml vs. ≤ 200 ml per 24 h). Residual urine output was assessed at study entry based on patient-reported 24-h urine volume and used as a stratification variable (>200 ml vs. ≤ 200 ml per 24 h). Randomization was performed centrally using an online tool (www.randomizer.at), and both participants and investigators remained blinded to treatment allocation. Study visits were scheduled at baseline and 6 months, with additional safety monitoring conducted at every dialysis session. These routine contacts were used to record adverse events, monitor blood chemistry (including glucose, pH, and potassium) as available according to local clinical practice, and assess drug tolerability and adherence. Echocardiographic assessments were scheduled at baseline and at 6 months and performed immediately after dialysis. Dialysis prescriptions, including ultrafiltration targets and dry weight, were determined at the discretion of the treating nephrologists in accordance with routine clinical practice.

Echocardiographic examinations were performed and analysed by a dedicated investigator (CM) with formal national certification in transthoracic echocardiography, who was blinded to treatment allocation. The same investigator conducted on-site image acquisition across participating centres and performed centralized analysis according to a standardized imaging protocol. Technically challenging cases were reviewed in conjunction with a second investigator (TAZ), who is board-certified in cardiology and holds national and EACVI certification in adult transthoracic echocardiography. At external participating sites, study-specific echocardiographic acquisitions were performed using the same ultrasound system (Canon Medical Systems), which was provided free of charge for study use and transported between sites. The manufacturer had no role in study design, data acquisition, analysis, interpretation, or manuscript preparation. Left ventricular mass was calculated using two-dimensional linear measurements obtained from the parasternal long-axis view at end-diastole in accordance with current ASE/EACVI guideline recommendations, using the Devereux-modified ASE formula and indexed to body surface area. Studies deemed non-evaluable due to insufficient image quality were classified as missing for the primary endpoint.

Additional assessments included laboratory analyses with biomarker serum samples obtained from the first blood draw at the beginning of the dialysis session, as well as quality of life evaluation using the Kidney Disease Quality of Life Short Form (KDQoL-SF, German version 1.2) at baseline and 6 months.^13^

### Study objectives and endpoints

The primary endpoint of DAPA-HD is the change in left ventricular mass indexed to body surface area (LVMI), as measured by echocardiography after 6 months of treatment. Originally, the primary endpoint was defined as the change in LVMI measured by cardiac magnetic resonance imaging (cMRI). Shortly after study initiation and prior to unblinding, the primary imaging modality was changed to transthoracic echocardiography due to feasibility considerations and resource constraints inherent to an investigator-initiated trial. This protocol amendment was approved by the institutional ethics committee, and a revised sample size calculation was conducted accordingly.

Additional echocardiographic outcomes of interest include changes in left ventricular ejection fraction (LVEF), left atrial diameter, and strain analysis of the left atrium and the left and right ventricles. Additional assessments include changes in blood pressure, heart rate, metabolic parameters (including HbA1c, insulin, c-peptide, glucagon, β-hydroxybutyrate, GLP-1, cortisol, growth hormone, and pyruvate), cardiovascular biomarkers (hsTnT and NT-proBNP), and quality of life. All hospitalizations and deaths were recorded throughout the study period. Safety monitoring was conducted at each dialysis visit, with particular attention to signs of euglycemic ketoacidosis, genital infections, and electrolyte disturbances.

A key exploratory objective of the trial was to assess whether the cardiovascular effects of dapagliflozin are modified by residual urine volume. A prespecified subgroup analysis will compare treatment effects between patients with residual urine output >200 ml/24 hours versus ≤200 ml/24 h.

### Statistical considerations

A sample size of 186 patients was calculated to provide 90% power to detect a 7 g/m^2^ difference in the primary endpoint at a two-sided alpha of 0.05, assuming a standard deviation of 15 g/m^2^. Accounting for an anticipated dropout rate of 20%, the target enrolment was set at 220 patients. The primary hypothesis will be tested using an analysis of covariance, adjusting for baseline LVMI. The primary analysis will use a complete-case approach, including all patients with available baseline and 6-month LVMI measurements. To assess the robustness of findings under alternative missing data assumptions, sensitivity analyses will be conducted using (i) multiple imputation assuming data are missing at random, with imputation models including baseline LVMI, treatment assignment, residual urine output stratum, age, sex, and diabetes status; and (ii) pattern-mixture models to evaluate potential bias under missing not at random scenarios, including differential dropout due to death or clinical deterioration. The extent and pattern of missing data will be reported descriptively by treatment group. In prespecified subgroup analyses, outcomes will be tested with interaction terms in the ANCOVA model to assess effect modification. Serious adverse events and study drug discontinuations will be summarized descriptively by treatment group.

### Current status and baseline characteristics

The trial completed enrolment and randomization of 220 patients with kidney failure with or without residual urine output between October 2022 and January 2025 across nine sites in Austria. The baseline characteristics of the study population are presented in *Table 2*. Overall, the median age of the study population is 67 years (Q1–Q3: 57–75 years), 26% of participants are women, 42% have type 2 diabetes mellitus, 39 % have established coronary artery disease, and 28 % of patients have residual urine output ≤200 ml/day. Baseline echocardiography showed a mean LVMI of 138 g/m^2^ (standard deviation 37 g/m^2^) and a median LVEF of 59 % (Q1–Q3: 54%–65%) with 10% having an LVEF ≤40%; the median NT-proBNP concentration was 2911 pg/ml (Q1–Q3: 1181–7602 pg/ml).

**Table 2:** Baseline characteristics of the overall DAPA-HD trial population (N = 220)

	Overall
Age, years (median, IQR)	67 (57–75)
Female sex, *n* (%)	57 (25.9%)
BMI, kg/m^2^ (mean ± SD)	28.2 (5.1)
Type 2 diabetes, *n* (%)	92 (41.8%)
LVEF ≤40%, *n* (%)	19 (9.7%)
Atrial fibrillation, *n* (%)	27 (12.3%)
Coronary artery disease, *n* (%)	86 (39.1%)
Hypertension, *n* (%)	193 (87.7%)
Presumed primary cause of kidney failure	
Diabetes, *n* (%)	58 (26.4%)
Hypertension, *n* (%)	23 (10.5%)
Polycystic, *n* (%)	20 (9.1%)
Nephritis, *n* (%)	32 (14.5%)
Other/Unknown	87 (39.5%)
Residual urine volume ≤200 ml/day, *n* (%)	62 (28.2%)
Baseline medication:	
ACEi/ARB, *n* (%)	112 (50.1%)
Beta-blockers, *n* (%)	160 (72.7%)
Loop diuretics, *n* (%)	111 (50.5%)
Insulin, *n* (%)	42 (19.1%)
MRA, *n* (%)	12 (5.5%)
Baseline echocardiographic measurement:	
LV mass, g (mean ± SD)	266 (77.0)
LV mass index, g/m^2^ (mean ± SD)	138 (36.7)
LVEF, % (median, IQR)	59 (54–65)
NT-proBNP, pg/ml (median, IQR)	2911 (1181–7602)

Abbreviations: IQR, interquartile range; SD, standard deviation; BMI, body mass index; ACEi/ARB, angiotensin-converting enzyme inhibitor/angiotensin II receptor blocker; MRA, mineralocorticoid receptor antagonist; LV, left ventricular; NT-proBNP, N-terminal pro-B-type natriuretic peptide

## Discussion

The DAPA-HD trial is the first randomized controlled trial to evaluate the cardiovascular effects of SGLT2i in patients with kidney failure receiving maintenance haemodialysis.

Cardiovascular disease remains the predominant cause of death among haemodialysis patients, with adverse cardiac remodelling developing in response to pressure and volume overload as a major contributor to adverse events.^4,14,15^ Chronic kidney disease drives a complex interplay of pathophysiological processes, including activation of the renin-angiotensin-aldosterone and sympathetic nervous systems, oxidative stress, systemic inflammation, and profibrotic signaling.^16–19^ These mechanisms, together with vascular calcification and endothelial dysfunction that increase arterial stiffness and cardiac afterload,^20^ and along with sodium and fluid retention, ultimately lead to ventricular hypertrophy and fibrosis. For patients with kidney failure undergoing haemodialysis, traditional cardioprotective therapies, including statins, have shown limited efficacy, reflecting a shift in pathophysiology toward nonatherosclerotic mechanisms such as left ventricular hypertrophy, arrhythmias, vascular calcification, and sudden cardiac death.^21,22^ Thus, preventing or reversing maladaptive cardiac remodelling is a major unmet therapeutic need in this population.^23^

Heart failure assessment in dialysis patients, however, is challenging due to the dynamic nature of volume overload and the limitations of conventional NYHA or AHA/ACC classifications in this population. The Acute Dialysis Quality Initiative (ADQI) XI work group, therefore, proposed a functional classification that integrates echocardiographic evidence of structural or functional cardiac abnormalities (e.g. left ventricular mass) and dyspnoea assessment before and after renal replacement therapy.^24^ DAPA-HD was thus designed to assess change in left ventricular mass index as the primary endpoint, a validated surrogate of cardiovascular risk and a sensitive marker of reverse remodeling.^23,24^ Stratification by residual urine volume will provide additional exploratory mechanistic insights into whether potential cardiac effects of dapagliflozin depend on residual kidney function.

SGLT2i confer cardiovascular benefits independent of glucose lowering and across a wide range of kidney functions, down to an eGFR of 20 ml/min/1.73 m^2^.^7,25^ Their multifaceted effects involve modest reductions in blood pressure and plasma volume, natriuresis, improved tubular-glomerular feedback, increased erythropoiesis, ketone body-mediated energetic shifts, decreased sympathetic activity, and reduction in inflammation, oxidative stress, and fibrosis.^26–28^ However, many of these effects are presumed to depend on residual kidney function and active sodium-glucose cotransport, raising questions about whether SGLT2i retain efficacy in the absence of glomerular filtration. Evidence regarding SGLT2i use in dialysis populations is currently limited to observational analyses and small pilot studies, which suggest acceptable short-term safety but are insufficient to establish cardiovascular efficacy due to inherent confounding and limited sample size.^29^ By integrating comprehensive echocardiographic assessments, biomarkers, safety monitoring, and quality-of-life measures, DAPA-HD is designed to provide comprehensive mechanistic and safety insights into the effects of dapagliflozin in patients with kidney failure receiving maintenance haemodialysis. The trial tests a central biological hypothesis that SGLT2i may confer cardiovascular benefits even in patients with complete anuria, implying kidney-independent mechanisms, potentially mediated by systemic metabolic, haemodynamic, or neurohumoral modulation. Either outcome will advance our understanding of SGLT2i biology and inform their use across the CKD-to-dialysis transition, and complement the ongoing event-driven Renal Lifecycle trial (NCT05374291), which evaluates dapagliflozin on cardiorenal outcomes, including kidney failure, heart-failure hospitalization, and all-cause mortality in 1500 patients across a broad spectrum of patients with CKD, including patients with an eGFR ≤25 ml/min/1.73 m2, those receiving haemodialysis or peritoneal dialysis and kidney transplant recipients, while also incorporating imaging substudies to assess cardiac structure and function.^30^

## Summary

In summary, the DAPA-HD trial (NCT05179668) is an academic, investigator-initiated, multicentre, randomized, double-blind, placebo-controlled phase 2 trial designed to evaluate the effects of dapagliflozin on cardiac structure and function in patients with kidney failure receiving maintenance haemodialysis.

## Declarations

### Data Availability

The data underlying this article will be shared on reasonable request to the corresponding author.
